# Supplementary material for: Male allyship to advance women’s leadership in global health academia: A qualitative study
Source: PLOS Glob Public Health. 2026 Apr 15;6(4):e0005258. doi: 10.1371/journal.pgph.0005258 (PMC13082618; doi:10.1371/journal.pgph.0005258)
Supplement: Attachment — Submitted filename: Manuscript ID PGPH-D-25-02685 Review Report.docx [file pgph.0005258.s003.docx]

Manuscript Number: PGPH-D-25-02685 Review Report Male Allyship

Overall summary of the review

Strength

This manuscript addresses the important topic of male allyship in advancing women’s leadership in global health academia, using a well-structured qualitative approach and presenting findings across individual, institutional, and societal levels. Strengths include clear objectives, rigorous methods, and practical insights for policy and leadership.

Areas that need improvement include:

- Clarify the study type in the title and make it action-oriented.
- Expand geographic representation beyond the U.S. and Canada.
- Provide context for participant quotes.
- Report data saturation and response rates.
- Improve readability by breaking up long sentences and structuring results around clear subthemes.
- acknowledge limitation for geographic representation beyond the U.S. and Canada

Point by point feedback

Title: - Male Allyship to Advance Women's Global Health Leadership in the Academy

**Strength**

- It addresses a timely and **high-Impact Topic**: The study area is forgotten by global community especially in academia on the importance of gender equity and leadership.
- The title accurately reflects the manuscript’s core theme male allyship in academic global health leadership.
- **Timely and High-Impact Topic:** The study addresses a significant gap in global health leadership literature—moving beyond simply identifying barriers to focusing on **actionable solutions** (allyship and sponsorship). This shift in focus is highly valuable for policymakers and institutional leaders

**Weakness**

- The short and long title of the manuscript is the same; no difference
- The title talks about two things one “Global Health Leadership” and second “the academic institution in North America”. the focus of study population is not clear
- The kind of the study type is no clearly indicated in the title.
- The phrase “in the Academy” could be slightly ambiguous to international audiences—consider specifying “in academic global health institutions.”

### Suggested Revision

- It is better if the title is an action oriented Like “Exploring Male Allyship to Advance Women’s Leadership in Global Health Academia: A Qualitative Study” or Male Allyship to Advance Women’s Leadership in Global Health Academia: A Qualitative Study

**Abstract**

**Strengths:**

**Introduction**

- Well-structured abstract with clear objectives, methods, and findings.
- Strong justification for the study, citing global gender disproportionate disparities and responsibilities gaps in global health leadership.
- **Appropriate Methodology:** It used a qualitative, semi structured interview approach to explore the perception, experiences and perceptions of high level leaders. Clear thematic analysis
- It use clear research question on the experience of global leaders
- **Findings**: Clearly structured around three levels—individual male ally, institutional, and societal level which offers a useful conceptual framework to shift cultural norms on gender roles for practical implications

**Area of improvement:**

- Introduction looks like an advocacy; no clearer separation between background and rationale.
- • Participants were drawn only from the U.S. and Canada, which limits the geographic coverage of the study. In addition, selecting participants exclusively from the WomenLift Health network may reduce the diversity of perspectives and potentially affect the credibility of the findings.
- **Conceptual clarity:** The term *male allyship* could be briefly defined in one sentence for clarity.
- The conclusion partially repeats ideas from the introduction it looks like the summary of the introduction and better if based on the findings and highlighting the practical or policy relevance to enhance the impact

Main Manuscript

- The study team approached participants via email and invited them to participate and conduct the interview via zoom; so why the study participants restricted to two high-income countries?

**Result**

- **Line 178-183**: The results section reports the number of participants but does not indicate whether data saturation was achieved. In qualitative research, explaining when and how saturation was reached strengthens the credibility and adequacy of the sample size.
- Reporting the response rate as a percentage would help readers interpret the level of participation more easily.
- **Line 185-191:** The paragraph communicates the general findings, better if key themes are mentioned to give readers a concrete examples
- **Line 202:** The quote is powerful, but providing brief context about the respondent role, experience, or institution better if included.
- Line Motivation and incentives it is more plausible to read if concrete example of p**articipant quotes are included**
- **Line 252 to 278:** the section contains valuable insights, but some sentences are long and dense. Breaking them into shorter sentences will improve readability. Consider structuring the section around clear subthemes: like awareness, role modeling etc.,
- Line 332-341 quote -P7, F, 40-44 is clear and conventional; however it’s a bit long and better if lightly edit for readability while retaining authenticity

# Discussion

### Strength

### It is strong in structure, logic, and scholarly tone. It clearly links findings to prior research and offers practical and theoretical insights

### Line 633 Toking et al reference number (54). Line 638 Sinha et al (59) need consistency

### Limitation

### Strength: Clearly states the population studied & acknowledges selection of diverse perspectives

**Area of Improvement:** Findings may not generalize to early-career leaders, non-academic settings, or global contexts.
