# Supplementary material for: Male allyship to advance women’s leadership in global health academia: A qualitative study
Source: PLOS Glob Public Health. 2026 Apr 15;6(4):e0005258. doi: 10.1371/journal.pgph.0005258 (PMC13082618; doi:10.1371/journal.pgph.0005258)
Supplement: Attachment — Submitted filename: Response to Reviewers.docx [file pgph.0005258.s004.docx]

December 18, 2025

Dear Dr. Noor,

Thank you for your feedback and review of our manuscript “Male Allyship to Advance Women's Global Health Leadership in the Academy” (PGPH-D-25-02685) to PLOS Global Public Health. We are pleased to submit a revised version of the manuscript that addresses the points raised by you and the other reviewers during the review process.

*This is an important article and, in my view, a strong contribution to the growing body of knowledge on male allyship in global health. The manuscript has been reviewed by two experts in the field, both of whom raised thoughtful concerns that I encourage the authors to address in their revisions.*

*From my perspective, the study offers potentially valuable insights, but several of them could be drawn out more clearly. Although you engage with concepts such as allyship and intersectionality, these ideas are currently discussed in a relatively narrow way. I recommend strengthening the conceptual grounding by engaging more deeply with existing literature on these concepts and offering a brief but clear conceptualisation early on. Doing so would help orient readers and better signal what to expect in the findings.*

Thank you for this positive feedback regarding our manuscript and your suggestions. We have incorporated the concepts of allyship and intersectionality into the paper’s introduction. The addition of both concepts has been informed by existing literature. See Expanding the Pool of Male Allies, lines 132-142 in the revised manuscript.

*Additionally, while you acknowledge that your sample does not include participants from several ethnic and gender identities, such as Middle Eastern, Southeast Asian, East Asian, Latin American, nonbinary, or transgender individuals, you should also expand on what perspectives these groups might have added. Reflecting on how their inclusion could have enriched your analysis and noting the need for further inquiry to understand how intersectionality operates in global health more broadly would enhance the transparency and reflexivity of the study. Some literature about inequalities faced by people with intersectional identities and those in LMICs could strengthen your manuscript and encourage research in this field.*

Had we been able to include additional ethnic, gender, and ability identities, the perspectives shared by the participants would have been more diverse and richer (see Limitations and Next Steps; lines 647-652). We may have also gathered additional insights into how allies do not always need to be a part of the dominant group as long as they are challenging existing power asymmetries and coupling this with action. As suggested, we have called for further research into the relationship of intersectionality and inequalities in global health in the discussion, see lines 657-667.

*Please include the following items when submitting your revised manuscript:*

- *A rebuttal letter that responds to each point raised by the editor and reviewer(s). You should upload this letter as a separate file labeled* ***'Response to Reviewers'****.*
- *A marked-up copy of your manuscript that highlights changes made to the original version. You should upload this as a separate file labeled* ***'Revised Manuscript with Track Changes****'.*
- *An unmarked version of your revised paper without tracked changes. You should upload this as a separate file labeled* ***'Manuscript'****.*

*Guidelines for resubmitting your figure files are available below the reviewer comments at the end of this letter.*

Please note we have updated S1 Table. We submitted a clean version and another with track changes.

*We look forward to receiving your revised manuscript.*

*Kind regards,*

*Naveed Noor, PhD*
*Academic Editor*
*PLOS Global Public Health*

*Journal Requirements:*

*1. Please send a completed 'Competing Interests' statement, including any COIs declared by your co-authors. If you have no competing interests to declare, please state "The authors have declared that no competing interests exist". Otherwise please declare all competing interests beginning with the statement "I have read the journal's policy and the authors of this manuscript have the following competing interests:"*

OA has declared that no competing interests exist. MB and AMC have read the journal's policy and have the following competing interests: MB is the founder and Honorary Board Member of WomenLift Health’s Global Advisory Board. AMC is a North American WomenLift Health Fellow.

*2. Please amend your detailed Financial Disclosure statement. This is published with the article. It must therefore be completed in full sentences and contain the exact wording you wish to be published.*

This work was supported WomenLift Health/New Venture Fund (NVF-STA25-WOLH1-2023-11-01 to MB). The funders provided input into the interview participant list and interview guide but otherwise had no role in data collection and analysis, decision to publish, or preparation of the manuscript. AMC and OA received partial salary support from WomenLift Health/New Venture Fund to conduct this research.

*i. Please clarify all sources of financial support for your study. List the grants, grant numbers, and organizations that funded your study, including funding received from your institution. Please note that suppliers of material support, including research materials, should be recognized in the Acknowledgements section rather than in the Financial Disclosure.* 
*ii. State the initials, alongside each funding source, of each author to receive each grant. For example: "This work was supported by the National Institutes of Health (####### to AM; ###### to CJ) and the National Science Foundation (###### to AM)."*
*iii. State what role the funders took in the study. If the funders had no role in your study, please state: “The funders had no role in study design, data collection and analysis, decision to publish, or preparation of the manuscript.”*
*iv. If any authors received a salary from any of your funders, please state which authors and which funders.*

*3. Please ensure that your Ethics Statement is available in its entirety at the beginning of your Methods section, under a subheading 'Ethics Statement'.*

We have revised the statement to be under the heading of “Ethics Statement” (lines 190-193) instead of “Ethical Considerations” and included the following: “This study was approved by the Stanford University Institutional Review Board (#72715). All participants received a copy of the consent form via email prior to the interview date, and 100% gave verbal consent before starting the interview.”

*4. In the online submission form, you indicated that “The data set is available upon request from the corresponding author.”*

*All PLOS journals now require all data underlying the findings described in their manuscript to be freely available to other researchers, either* 
*1. In a public repository,* 
*2. Within the manuscript itself, or* 
*3. Uploaded as supplementary information.*

*This policy applies to all data except where public deposition would breach compliance with the protocol approved by your research ethics board. If your data cannot be made publicly available for ethical or legal reasons (e.g., public availability would compromise patient privacy), please explain your reasons by return email and your exemption request will be escalated to the editor for approval. Your exemption request will be handled independently and will not hold up the peer review process, but will need to be resolved should your manuscript be accepted for publication. One of the Editorial team will then be in touch if there are any issues.*

We are revising our statement about data availability and request an exemption. Despite de-identifying the data, it cannot be made publicly available for ethical and legal reasons (e.g., public availability would compromise participant privacy) as data could reveal the identities of participants.

*5. Some material included in your submission may be copyrighted. According to PLOS’s copyright policy, authors who use figures or other material (e.g., graphics, clipart, maps) from another author or copyright holder must demonstrate or obtain permission to publish this material under the Creative Commons Attribution 4.0 International (CC BY 4.0) License used by PLOS journals. Please closely review the details of PLOS’s copyright requirements here: PLOS Licenses and Copyright. If you need to request permissions from a copyright holder, you may use PLOS's Copyright Content Permission form.*

*Please respond directly to this email or email the journal office and provide any known details concerning your material's license terms and permissions required for reuse, even if you have not yet obtained copyright permissions or are unsure of your material's copyright compatibility.*

*Potential Copyright Issues:*

*Figures 1 and 2: Please confirm whether you drew the images / clip-art within the figure panels by hand. If you did not draw the images, please provide (a) a link to the source of the images or icons and their license / terms of use; or (b) written permission from the copyright holder to publish the images or icons under our CC-BY 4.0 license. Alternatively, you may replace the images with open source alternatives. See these open source resources you may use to replace images / clip-art:*

*-* [*https://commons.wikimedia.org*](https://commons.wikimedia.org)
*-* [*https://openclipart.org/*](https://openclipart.org/)

*If the reviewer comments include a recommendation to cite specific previously published works, please review and evaluate these publications to determine whether they are relevant and should be cited. There is no requirement to cite these works unless the editor has indicated otherwise.* 

Icons in Figures 1 and 2 are original art created by a graphic designer, Bhabna Banerjee, specifically for this research paper. We have obtained written permission to use and publish the figures and have submitted this with our revised manuscript.

*[Note: HTML markup is below. Please do not edit.]*

*Reviewers' comments:*

*Reviewer's Responses to Questions*

***Comments to the Author***

*1. Does this manuscript meet PLOS Global Public Health’s* [*publication criteria*](https://urldefense.com/v3/__https:/track.editorialmanager.com/CL0/https:*2F*2Fjournals.plos.org*2Fglobalpublichealth*2Fs*2Fcriteria-for-publication/1/010f019a9f15c10d-827791fe-a19d-416f-b4a3-1f0843e51a2c-000000/nc1prp6ObepuPk_EVMJN-HRSTV3O5zZj5e2NlxkdLNs=238__;JSUlJSU!!G92We9drHetJ8EofZw!c4Opd6UJtMaz3QCT-UHATnYnFdGtogi9xtgzWSImE88Zu0AL9jO4WAsULU0xvs-rlfwufmtvBJ0o5sc7GQ$)*? Is the manuscript technically sound, and do the data support the conclusions? The manuscript must describe methodologically and ethically rigorous research with conclusions that are appropriately drawn based on the data presented.*

*Reviewer #1: Yes*

*Reviewer #2: Yes*

*2. Has the statistical analysis been performed appropriately and rigorously?*

*Reviewer #1: N/A*

*Reviewer #2: N/A*

*3. Have the authors made all data underlying the findings in their manuscript fully available (please refer to the Data Availability Statement at the start of the manuscript PDF file)?*

*The* [*PLOS Data policy*](https://urldefense.com/v3/__https:/track.editorialmanager.com/CL0/https:*2F*2Fjournals.plos.org*2Fglobalpublichealth*2Fs*2Fdata-availability/1/010f019a9f15c10d-827791fe-a19d-416f-b4a3-1f0843e51a2c-000000/yZqmp1_INzW9jWnPuXAiOq9JF0wkwwozunbshYVCFE0=238__;JSUlJSU!!G92We9drHetJ8EofZw!c4Opd6UJtMaz3QCT-UHATnYnFdGtogi9xtgzWSImE88Zu0AL9jO4WAsULU0xvs-rlfwufmtvBJ1b6HBuLw$) *requires authors to make all data underlying the findings described in their manuscript fully available without restriction, with rare exception. The data should be provided as part of the manuscript or its supporting information, or deposited to a public repository. For example, in addition to summary statistics, the data points behind means, medians and variance measures should be available. If there are restrictions on publicly sharing data—e.g. participant privacy or use of data from a third party—those must be specified.*

*Reviewer #1: No*

*Reviewer #2: No*

As indicated above, we cannot make the data publicly available, as doing so would violate participant privacy.

*4. Is the manuscript presented in an intelligible fashion and written in standard English?*

*PLOS Global Public Health does not copyedit accepted manuscripts, so the language in submitted articles must be clear, correct, and unambiguous. Any typographical or grammatical errors should be corrected at revision, so please note any specific errors here.*

*Reviewer #1: Yes*

*Reviewer #2: Yes*

*5. Review Comments to the Author*

*Please use the space provided to explain your answers to the questions above. You may also include additional comments for the author, including concerns about dual publication, research ethics, or publication ethics. (Please upload your review as an attachment if it exceeds 20,000 characters)*

**Reviewer #1: Manuscript Number: PGPH-D-25-02685 Review Report Male Allyship**
**Overall summary of the review**
**Strength**
*This manuscript addresses the important topic of male allyship in advancing women’s leadership in global health academia, using a well-structured qualitative approach and presenting findings across individual, institutional, and societal levels. Strengths include clear objectives, rigorous methods, and practical insights for policy and leadership.*
***Areas that need improvement include:***
*• Clarify the study type in the title and make it action-oriented.*
*• Expand geographic representation beyond the U.S. and Canada.*
*• Provide context for participant quotes.*
*• Report data saturation and response rates.*
*• Improve readability by breaking up long sentences and structuring results around clear subthemes.*
*• acknowledge limitation for geographic representation beyond the U.S. and Canada*

We will address the areas needing improvement in responses to the point by point feedback below.

***Point by point feedback***

*Title: - Male Allyship to Advance Women's Global Health Leadership in the Academy*
***Strength***
*• It addresses a timely and high-Impact Topic: The study area is forgotten by global community especially in academia on the importance of gender equity and leadership.*
*• The title accurately reflects the manuscript’s core theme male allyship in academic global health leadership.*
*• Timely and High-Impact Topic: The study addresses a significant gap in global health leadership literature—moving beyond simply identifying barriers to focusing on actionable solutions (allyship and sponsorship). This shift in focus is highly valuable for policymakers and institutional leaders*
***Weakness***
*• The short and long title of the manuscript is the same; no difference*
*• The title talks about two things one “Global Health Leadership” and second “the academic institution in North America”. the focus of study population is not clear*
*• The kind of the study type is no clearly indicated in the title.*
*• The phrase “in the Academy” could be slightly ambiguous to international audiences—consider specifying “in academic global health institutions.”*
***Suggested Revision***
*• It is better if the title is an action oriented Like “Exploring Male Allyship to Advance Women’s Leadership in Global Health Academia: A Qualitative Study” or Male Allyship to Advance Women’s Leadership in Global Health Academia: A Qualitative Study*

Thank you for the positive feedback above. We have revised the title to the latter suggestion.

***Abstract***
***Strengths:***
*Introduction*
*• Well-structured abstract with clear objectives, methods, and findings.*
*• Strong justification for the study, citing global gender disproportionate disparities and responsibilities gaps in global health leadership.*
*• Appropriate Methodology: It used a qualitative, semi structured interview approach to explore the perception, experiences and perceptions of high level leaders. Clear thematic analysis*

*• It use clear research question on the experience of global leaders*
*• Findings: Clearly structured around three levels—individual male ally, institutional, and societal level which offers a useful conceptual framework to shift cultural norms on gender roles for practical implications*
**Area of improvement:**
*• Introduction looks like an advocacy; no clearer separation between background and rationale.*

We have revised the manuscript to clearly delineate the background from the rationale for the study in the introduction, see Expanding the Poll of Male Allies section, line 131.

• *Participants were drawn only from the U.S. and Canada, which limits the geographic coverage of the study. In addition, selecting participants exclusively from the WomenLift Health network may reduce the diversity of perspectives and potentially affect the credibility of the findings.*

We acknowledge the geographical limitations on our findings but were restricted by our funding. Based on our positionality in the U.S. we concluded that local investigators would be better placed to lead additional research in other geographies. As we mentioned in the Methods section, in addition to the WomenLift Health list, we relied on the PI and CoPI’s networks, snowball sampling to expand the list, and searching the directories of academic and clinical institutions with a global health focus for faculty who have extensive experience working in global health. We expanded on the Methods section to include more information on recruitment (lines 158-162).

*• Conceptual clarity: The term male allyship could be briefly defined in one sentence for clarity.*

This term was defined in the Methods section, table 1 (line 166): “Purposeful collaboration of dominant group having power and privilege, men, with subordinate group having less power and privilege, women, to achieve gender equity by promoting women’s voices and accomplishments.”

*• The conclusion partially repeats ideas from the introduction it looks like the summary of the introduction and better if based on the findings and highlighting the practical or policy relevance to enhance the impact*

We have revised the conclusion so that it does not repeat ideas from the introduction and highlights the implication of our findings, including the need for more research related to intersectionality and gender inequities, public education, advocacy, and changes to policy and practice within institutions, see lines 656-667.

**Main Manuscript**
*• The study team approached participants via email and invited them to participate and conduct the interview via zoom; so why the study participants restricted to two high-income countries?*

We were restricted by our funding and also acknowledge our positionality as being based in a HIC, with LMIC researchers being better placed to conduct further research in their respective geographies. We will be collaborating with local researchers to expand engagement across other regions through focus groups and a global survey. We amended the Limitations (lines 642-647) and Next Steps sections (lines 658-661).

***Result***
*• Line 178-183: The results section reports the number of participants but does not indicate whether data saturation was achieved. In qualitative research, explaining when and how saturation was reached strengthens the credibility and adequacy of the sample size.*

Thank you for pointing this out. We have amended the Methods section to address data saturation, see lines 177-179.

*• Reporting the response rate as a percentage would help readers interpret the level of participation more easily.*

We have included a response rate percentage as suggested in our Results section, see lines 196-197.

*• Line 185-191: The paragraph communicates the general findings, better if key themes are mentioned to give readers a concrete examples*

We have revised this paragraph to refer to Figure 1, which outlines the key themes, as suggested (lines 208-210).

*• Line 202: The quote is powerful, but providing brief context about the respondent role, experience, or institution better if included.*

We added additional context about the respondent’s experience and also corrected the quote that they shared with us (lines 225-229).

*• Line Motivation and incentives it is more plausible to read if concrete example of participant quotes are included*

We have included a relevant quote from a participant to illustrate this point (see lines 253-257).

*• Line 252 to 278: the section contains valuable insights, but some sentences are long and dense. Breaking them into shorter sentences will improve readability. Consider structuring the section around clear subthemes: like awareness, role modeling etc.,*

We have revised this section to break long sentences into shorter ones (lines 261-279). We did not break it into further subthemes as the 2^nd^ reviewer advised us to cut down on the number of subthemes.

*• Line 332-341 quote -P7, F, 40-44 is clear and conventional; however it’s a bit long and better if lightly edit for readability while retaining authenticity*

As suggested, we have cut this quote down to improve readability (lines 345-350).

**Discussion**
**Strength**
• *It is strong in structure, logic, and scholarly tone. It clearly links findings to prior research and offers practical and theoretical insights*
*• Line 633 Toking et al reference number (54). Line 638 Sinha et al (59) need consistency*

We addressed this (lines 631-632 and 636-638).

**Limitation**
***Strength:*** *Clearly states the population studied & acknowledges selection of diverse perspectives*
***Area of Improvement:*** *Findings may not generalize to early-career leaders, non-academic settings, or global contexts.*

We have revised the Limitations section to make it clear that our findings may not be generalizable to these groups and contexts (lines 642-643).

*Reviewer #2: This paper examines an important topic: male allyship in global health. A key strength of the paper is its focus on solutions as reported by participants. This helps move the focus away from challenges women in global health face, to exploring actual solutions to the problem. The paper presents many examples of what male allyship looks like, many of these being very actionable. I commend the authors for this. That said, there are several areas that need attention.*

*1. The findings could be synthesized and made more concise. The authors present A LOT of information, which is overwhelming. There are many ways to address this. First, the authors can use much shorter direct excerpts from participants. Second, the authors could move participant excerpts into a table. Third, the authors could synthesize their findings much more which would reduce the number of themes/issues and tell the story a bit differently. The paper would read better if they structured the results based on the subheadings used in Fig 1. It would help tell a more succinct story.*

We appreciate the feedback and have shortened quotes where possible. We chose not to move quotes into a table as readers may lose the context when separated from the relevant text of the findings. We have also consolidated the themes/issues in the results using the subheadings in Figure 1 as suggested.

*2. It is not clear what Fig 2 is showing.*

Participants shared examples of policies that institutions could adopt to advance gender equity. Figure 2 serves to illustrate the main categories and examples of such policies.

*3. I wonder if to would help to present the findings by gender, that is, by showing similarities and differences between female and male participants.*

While we could have compared and contrasted our findings by gender, our aim was to focus on recommendations, resources, and concrete actions to lift up women leaders, as challenges have been studied and documented before. With the exception of biological differences between the sexes, such as understanding the impact of menstruation and menopause on women and having policies to accommodate them, there weren’t differences in recommendations between males and females. We added this to the Results section lines 214-217.

*4. Could the authors present more details on the study participants. Sone of the content in the excerpts suggests that they were from academic institutions. A demographics table would help, in terms of academic vs non-academic institutions etc. The authors should also provide more details on the sampling and recruitment method: how exactly did they find these 21 participants?*

We have added a demographics table (Table 2) under the Results section (lines 202-206). All of our participants were connected to an academic institution. As mentioned in the Methods section, we populated a list of potential participants based on our connections and knowledge of leaders in global health and then used snowball sampling to expand this list. Additionally, we searched directories of academic and clinical institutions that have a global health focus for faculty who have extensive experience working in global health. We revised the Methods section to include more information on recruitment (lines 158-162).

*5. The Discussion could be strengthened by a more critical analysis of the concept of male allyship. The authors should consider what their findings/study implications are for the potential and pitfalls of the concept.*

We have revised our discussion to emphasize the important role that women, including those from marginalized groups, can play in elevating themselves and other women. We also acknowledged the need for women and other underrepresented groups to bolster this movement in the last paragraph of discussion (lines 663-667).

*6. PLOS authors have the option to publish the peer review history of their article (*[*what does this mean?*](https://urldefense.com/v3/__https:/track.editorialmanager.com/CL0/https:*2F*2Fjournals.plos.org*2Fglobalpublichealth*2Fs*2Feditorial-and-peer-review-process*23loc-peer-review-history/1/010f019a9f15c10d-827791fe-a19d-416f-b4a3-1f0843e51a2c-000000/Z5jJlyDT_fhQYx2h3jL5pJVK3wElR5F-xjIySfT-r4c=238__;JSUlJSUl!!G92We9drHetJ8EofZw!c4Opd6UJtMaz3QCT-UHATnYnFdGtogi9xtgzWSImE88Zu0AL9jO4WAsULU0xvs-rlfwufmtvBJ17n6gsxQ$)*). If published, this will include your full peer review and any attached files.*


***Do you want your identity to be public for this peer review?*** *If you choose “no”, your identity will remain anonymous but your review may still be made public.*


*For information about this choice, including consent withdrawal, please see our* [*Privacy Policy*](https://urldefense.com/v3/__https:/track.editorialmanager.com/CL0/https:*2F*2Fwww.plos.org*2Fprivacy-policy/1/010f019a9f15c10d-827791fe-a19d-416f-b4a3-1f0843e51a2c-000000/T7Gq0_5XoNomwW68KYTMxBdnRSqcLfEcaqFAE3Ub-Yc=238__;JSUl!!G92We9drHetJ8EofZw!c4Opd6UJtMaz3QCT-UHATnYnFdGtogi9xtgzWSImE88Zu0AL9jO4WAsULU0xvs-rlfwufmtvBJ2Vi02kdQ$)*.*

*Reviewer #1: No*

*Reviewer #2:* ***Yes:*** *Tsitsi B Masvawure*

*[NOTE: If reviewer comments were submitted as an attachment file, they will be attached to this email and accessible via the submission site. Please log into your account, locate the manuscript record, and check for the action link "View Attachments". If this link does not appear, there are no attachment files.]*

*Figure Resubmissions:*

*While revising your submission, we strongly recommend that you use PLOS’s NAAS tool (https://ngplosjournals.pagemajik.ai/artanalysis) to test your figure files. NAAS can convert your figure files to the TIFF file type and meet basic requirements (such as print size, resolution), or provide you with a report on issues that do not meet our requirements and that NAAS cannot fix.*

*After uploading your figures to PLOS’s NAAS tool -* [*https://ngplosjournals.pagemajik.ai/artanalysis*](https://ngplosjournals.pagemajik.ai/artanalysis) *, NAAS will process the files provided and display the results in the "Uploaded Files" section of the page as the processing is complete. If the uploaded figures meet our requirements (or NAAS is able to fix the files to meet our requirements), the figure will be marked as "fixed" above. If NAAS is unable to fix the files, a red "failed" label will appear above. When NAAS has confirmed that the figure files meet our requirements, please download the file via the download option, and include these NAAS processed figure files when submitting your revised manuscript.*

Thank you for this suggestion. We tried this, but it lowered the resolution of our figures.
